# Quality control ensures fidelity in ribosome assembly and cellular health

**DOI:** 10.1083/jcb.202209115

**Published:** 2023-02-15

**Authors:** Melissa D. Parker, Katrin Karbstein

**Affiliations:** 1 The Skaggs Graduate School of Chemical and Biological Sciences, The Scripps Research Institute, La Jolla, CA, USA; 2 University of Florida—Scripps Biomedical Research, Jupiter, FL, USA; 3 Howard Hughes Medical Institute Faculty Scholar, Howard Hughes Medical Institute, Chevy Chase, MD, USA

## Abstract

The coordinated integration of ribosomal RNA and protein into two functional ribosomal subunits is safeguarded by quality control checkpoints that ensure ribosomes are correctly assembled and functional before they engage in translation. Quality control is critical in maintaining the integrity of ribosomes and necessary to support healthy cell growth and prevent diseases associated with mistakes in ribosome assembly. Its importance is demonstrated by the finding that bypassing quality control leads to misassembled, malfunctioning ribosomes with altered translation fidelity, which change gene expression and disrupt protein homeostasis. In this review, we outline our understanding of quality control within ribosome synthesis and how failure to enforce quality control contributes to human disease. We first provide a definition of quality control to guide our investigation, briefly present the main assembly steps, and then examine stages of assembly that test ribosome function, establish a pass–fail system to evaluate these functions, and contribute to altered ribosome performance when bypassed, and are thus considered “quality control.”

## Teaser

Melissa Parker and Katrin Karbstein review our current understanding of the quality control mechanisms governing ribosome assembly.

## Introduction

As molecular machines essential for maintaining protein homeostasis, ribosomes are not only responsible for protein production but also play vital roles in protein and mRNA quality control (QC; Brandman et al., 2012; Bengtson and Joazeiro, 2010; Inada, 2020; He and Jacobson, 2015; D’Orazio and Green, 2021; Simms et al., 2017; Kim and Zaher, 2022). To support actively growing cells, thousands of ribosomes must be accurately constructed every minute. This process is dependent on nutrient availability, affected by stress and the cell cycle (Warner, 1999; Mahajan, 1994; Ju and Warner, 1994; Powers and Walter, 1999), and is promoted by over 200 transiently binding assembly factors (AFs) that ensure the production of the eukaryotic ribosome from the nucleolus through the nucleus into the cytoplasm. Assembly involves the transcription of four ribosomal RNAs (rRNAs), which are then modified, processed, folded, and bound to 79 ribosomal proteins (RPs; Woolford and Baserga, 2013; Bohnsack and Bohnsack, 2019) to produce two ribosomal subunits: the small 40S subunit and the large 60S subunit. Together, these subunits form the translationally competent 80S eukaryotic ribosome.

Considering that ribosome assembly commands a significant amount of cellular resources (Warner, 1999) and that most RPs are essential for proper ribosome function (de la Cruz et al., 2015), it is no surprise that cells would protect their investment by establishing QC checkpoints during ribosome assembly to ensure that each ribosomal component is correctly assembled and that ribosomes are fully functional before initiating translation.

Mutations in the assembly machinery can allow for bypass of QC checkpoints, thereby releasing impaired ribosomes for translation (Ghalei et al., 2017; Collins et al., 2018; Parker et al., 2019; Huang et al., 2020; Sulima et al., 2014b; Bussiere et al., 2012; Weis et al., 2015; De Keersmaecker et al., 2013). Misassembled ribosomes can alter protein homeostasis by mistranslating the genetic code, by shifting the protein output between different mRNAs via alteration of mRNA or start-codon selection, by increased frameshifting, which leads to non-sense-mediated decay, or by inviting collisions on well-translated mRNAs, leading to their degradation (Ghalei et al., 2017; Collins et al., 2018; Parker et al., 2019; Huang et al., 2020; Bussiere et al., 2012; Sulima et al., 2014b; Tye et al., 2019; Ferretti et al., 2017; De Keersmaecker et al., 2013). Haploinsufficiency of or mutations in RPs and AFs are causative for a group of diseases, collectively called ribosomopathies, that lead to dysregulated ribosome concentration and/or composition and increase the patient’s risk of developing cancer (Burwick et al., 2011; Farrar et al., 2011; Bolze et al., 2013; Armistead and Triggs-Raine, 2014; Vlachos et al., 2012; Freed et al., 2010; Amsterdam et al., 2004). It is important to note that both altered ribosome composition and altered numbers of ribosomes can change the translational output of a cell (Ivanov et al., 2022; Khajuria et al., 2018; Kondrashov et al., 2011; Lodish, 1974; Mills and Green, 2017; Ferretti and Karbstein, 2019). Conversely, the uniform expression of RPs is disrupted in several cancers and is associated with a poor prognosis (Guimaraes and Zavolan, 2016; Kulkarni et al., 2017; Vlachos, 2017). Intriguingly, loss of stoichiometry is found typically in p53 null cancers (Ajore et al., 2017), presumably because the checkpoints for free RPs that otherwise help impose stoichiometry (Zhang et al., 2003; Bursać et al., 2012; Oršolić et al., 2020; Lindström et al., 2022) are now inactivated. Together, these data indicate that QC during ribosome assembly is vital for maintaining the quality of ribosomes required for healthy cell growth while demonstrating the pressure on cells to maintain ribosome numbers, which could lead to the leaky checkpoints we describe below.

In this review, we discuss ribosome assembly through the lens of QC. As more is understood about how ribosomes are assembled and quality controlled in yeast than in mammalian cells (Henras et al., 2015; Bohnsack and Bohnsack, 2019), this review will focus on QC steps identified in yeast ribosome assembly, with links drawn to QC defects associated with human disease. We begin by providing a definition for QC to clarify our understanding of the term for this review and hopefully beyond. We then briefly cover the main assembly steps and dive in-depth into eight examples of QC mechanisms that drive accurate ribosome assembly.

## A definition of QC

The last two decades of research have widely expanded our molecular and structural understanding of ribosome assembly and uncovered many mechanisms that promote correct assembly. As not everything that enables accuracy in assembly is QC, which is typically understood to be a retroactive process (Inada, 2020; Jakubowski, 2012; Whipple et al., 2011; Jin et al., 2005; Pape et al., 1999), we first provide a definition for QC. It is hoped that this definition will clarify our use of the word for this work and promote a more unified use of the term in the future.

We consider three elements necessary to demonstrate a QC mechanism. (i) An assembly step must have a role in testing functionality. This could be as simple as being able to recruit a ligand of the mature ribosome to establish structural integrity or as complex as being able to carry out functions of the ribosome, such as conformational transitions or even peptidyl transfer. (ii) A pass–fail system must be established by which functional intermediates pass and are allowed to progress to the next assembly step while dysfunctional misassembled ribosomes are detained until proper assembly steps are taken or they are committed to degradation. In this way, QC prevents immature or misassembled ribosomes from participating in translation, ensuring that all translating ribosomes are functional. (iii) Finally, to call any such mechanism QC, we consider it essential to demonstrate that bypassing these mechanisms will increase the amount of improperly assembled ribosomes and/or abnormal ribosome function.

For example, hierarchical rRNA assembly steps can enable correct ribosome biogenesis by promoting proper folding and simplifying the assembly landscape. An example is the completion of previous nucleolar assembly steps, including the binding of RPs for the recruitment of nuclear export factors, as reported for the 40S AF RIOK2 in mammalian cells (Zemp et al., 2009). While this mechanism ensures that only intermediates containing Rps3 (uS3) are released into the cytoplasm, this is a passive attendance check that does not test functionality. A more ambiguous example is the recruitment of the 60S export factor Arx1, which binds the polypeptide exit tunnel and is thus poised to test whether the exit tunnel has been properly assembled (Greber et al., 2012). Nonetheless, it has not been demonstrated that mutants defective in exit channel function are defective in Arx1 binding. Thus, while this may end up being a QC mechanism, full evidence for QC is currently lacking. As few published examples feature all these requirements, including the demonstration of ribosome misassembly upon bypass, we present some examples that fit only the first two criteria.

## Ribosome assembly at a glance

**Figure 1.**
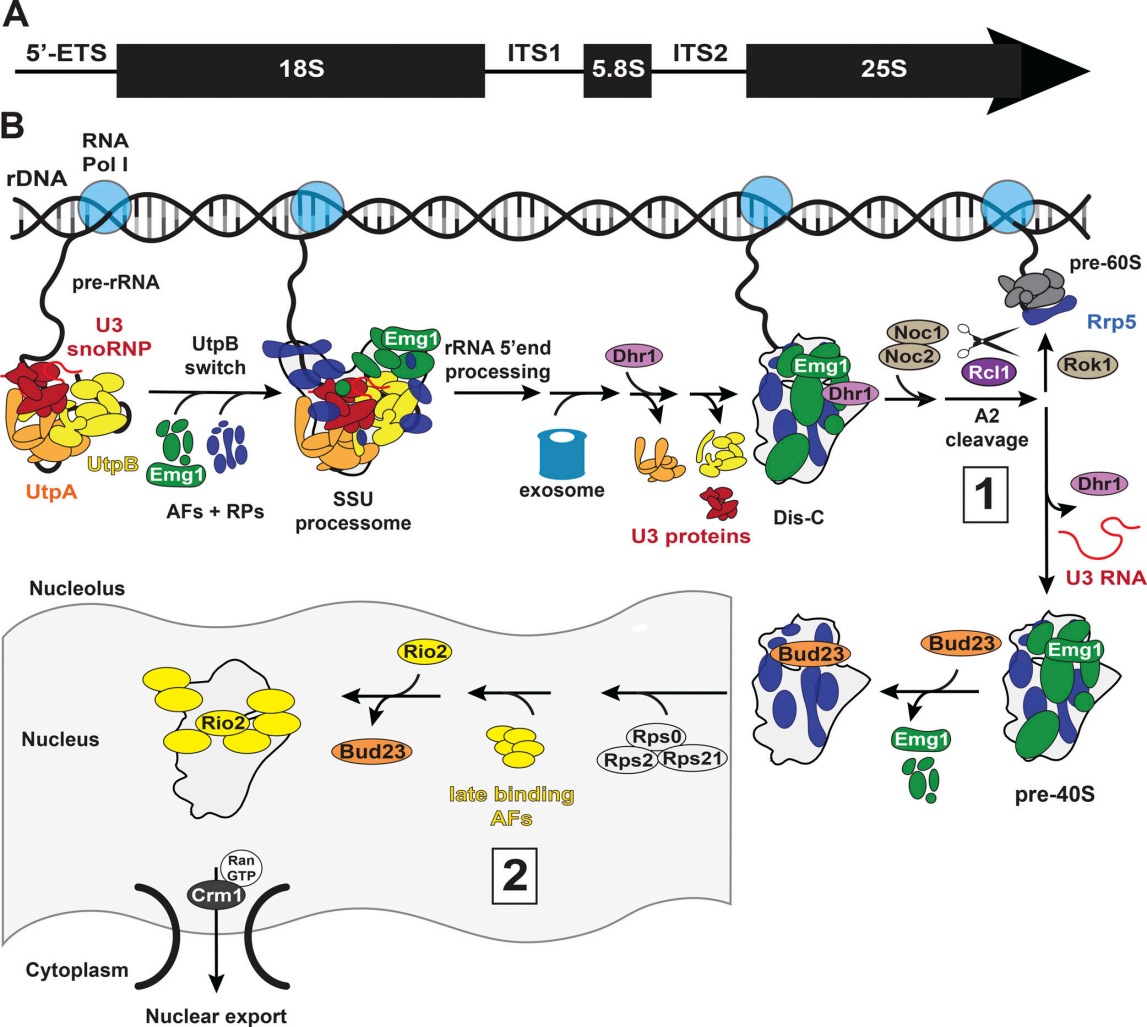
**Cartoon overview of SSU processome and nuclear pre-40S assembly. (A)** Schematic of the pre-rRNA encoding 18S, 5.8S, and 25S rRNAs. **(B)** Numbers 1–2 represent two potential QC steps: (1) balancing 40S:60S subunit production and (2) maturation of the ribosome neck. Described in more detail in the section titled “Nucleolar and nuclear pre-40S assembly.”

A brief outline of ribosome assembly is presented here to guide the discussion. Excellent in-depth reviews of the process include Henras et al. (2015); Woolford and Baserga (2013); Greber (2016); Konikkat and Woolford (2017); Pena et al. (2017); Barandun et al. (2018); Klinge and Woolford (2019); Bassle and Hurt (2019); Bohnsack and Bohnsack (2019). The life of a ribosome begins with the RNA polymerase I–driven transcription of a polycistronic precursor rRNA (pre-rRNA), encoding three of the four rRNAs (18S, 5.8S, and 25S or 28S rRNA), separated by external and internal transcribed spacers (5′ external transcribed spacer [5′-ETS], ITS1, ITS2, and 3′-ETS; Fig. 1 A), which are removed through multiple cleavage events during assembly. Simultaneously, RNA polymerase III transcribes the large ribosomal subunit 5S rRNA precursor and RNA polymerase II generates the mRNAs encoding RPs and the 200 AFs, which promote assembly, regulation, and QC.

### Nucleolar and nuclear pre-40S assembly

Assembly of the nascent 40S subunit occurs co-transcriptionally and in a hierarchical manner in the nucleolus (Pérez-Fernández et al., 2007; Pérez-Fernández et al., 2011; Hunziker et al., 2019; Chaker-Margot et al., 2015; Zhang et al., 2016; Fig. 1 B). Transcription of the 5′-ETS enables the recruitment of the UtpA protein complex, which recruits the UtpB protein complex. The UtpB complex serves as a platform for U3 small nucleolar RNA (snoRNA) base pairing with the 5′-ETS (Hunziker et al., 2016). This allows U3 to subsequently form additional base pairs with 18S rRNA that prevent premature folding of the central pseudoknot (Kudla et al., 2011; Dutca et al., 2011; Beltrame and Tollervey, 1992; Beltrame et al., 1994; Beltrame and Tollervey, 1995), a tertiary RNA structure that coordinates 40S subunit structure and comprises the decoding center (Vila et al., 1994). Upon completion of 18S rRNA transcription, the UtpB complex undergoes a conformational switch to enable the recruitment of late-binding AFs (Hunziker et al., 2019), thereby forming the so-called 90S pre-ribosome (also referred to as the small subunit processosome [SSU processosome]; Kornprobst et al., 2016; Hunziker et al., 2019; Sun et al., 2017; Barandun et al., 2017; Cheng et al., 2017), and ultimately licensing the first rRNA processing step. Domain compactions and 5′-ETS remodeling reposition the 5′-end of 18S rRNA into the active site of the nuclease (Utp24) that generates the mature 5′-end of 18S rRNA (Bleichert et al., 2006; Wells et al., 2016; Tomecki et al., 2015; Khoshnevis et al., 2019; Du et al., 2020; Cheng et al., 2020). The 5′-ETS can now be degraded by the nuclear exosome, ultimately leading to the release of Sof1, Utp7, and Utp6 (Cheng et al., 2020; Du et al., 2020; Lau et al., 2021), and weakening of Utp20. UtpA and the U3-associated proteins are released next, followed by release of UtpC and UtpB to form a 40S-like intermediate termed Dis-C (Cheng et al., 2020). Release of UtpB leads to repositioning of the DExH helicase Dhr1 (Cheng et al., 2020; Singh et al., 2021), enabling it to reach the U3 snoRNA and pull it out of the nascent ribosome. Moreover, Dis-C also appears to be the substrate for the methyltransferase Bud23, whose binding is associated with the release of the remaining nucleolar AFs (Black et al., 2020; Cheng et al., 2022).

These early assembly events occur co-transcriptionally until the nascent 60S is separated from the partially assembled 40S subunit via endonucleolytic cleavage (Osheim et al., 2004; Kos and Tollervey, 2010; Ismail et al., 2022). This appears to occur either within the Dis-C intermediate or just prior to its formation, as this intermediate has been processed (Cheng et al., 2020). Nevertheless, this separation requires not just 40S assembly but the transcription of 25S rRNA domain I (Kos and Tollervey, 2010; Osheim et al., 2004). The newly transcribed 25S rRNA is then bound by the pre-60S AFs Noc1/Noc2 (Khoshnevis et al., 2019), which in turn binds Rrp5 (Khoshnevis et al., 2019; Hierlmeier et al., 2013). This induces conformational changes within Rrp5 (Khoshnevis et al., 2019) that stimulate the Rcl1-dependent (Billy et al., 2000; Horn et al., 2011; Wells et al., 2016; Khoshnevis et al., 2019) endonucleolytic separation of the pre-40S and pre-60S subunits by cleavage between 18S and 5.8S rRNAs.^1^ During the final nuclear maturation steps, additional RPs and AFs required for export and cytoplasmic maturation bind pre-40S, which is then exported to the cytoplasm by the exportin Crm1 in a RanGTP-dependent manner (Thomas and Kutay, 2003; Fischer et al., 2015).

### Potential QC steps during nucleolar pre-40S maturation

Due to the labile nature of these early intermediates, our understanding of the mechanisms regulating nuclear assembly of the pre-40S subunit and its QC remains incomplete. Thus, there are currently no examples of nuclear assembly steps that meet all our criteria for QC. Nonetheless, we present two examples that meet two of the criteria but lack the characterization of effects from bypass. We have included those here to demonstrate where future research could go.

#### Maintaining 40S:60S subunit balance (Fig. 1, step 1)

Cleavage between 18S and 5.8S rRNAs separates the two nascent subunits to proceed through separate maturation pathways. Yet, it is vital that the ratio of 40S and 60S subunits is maintained as an imbalance changes gene expression and inhibits protein synthesis, slows cell growth, and in the case of excess 40S, leads to turnover of the excess subunit (Gregory et al., 2019; Cheng et al., 2019). To ensure the balanced production of both subunits, Rrp5 establishes a pass–fail checkpoint prior to this rRNA cleavage (Khoshnevis et al., 2019). One of only three AFs required for assembly of both subunits, Rrp5, a component of the UtpC complex, binds not only on the assembling platform (Kornprobst et al., 2016; Cheng et al., 2017; Sun et al., 2017) but also to ITS1 near the site of endonucleolytic separation (Young and Karbstein, 2011; Lebaron et al., 2013), thereby blocking this cleavage step (Khoshnevis et al., 2019). However, the interaction between Rrp5 and the rRNA is temporally regulated such that once domain I of 25S rRNA is transcribed, the pre-60S Noc1/Noc2 complex binds the rRNA and Rrp5, inducing a conformational change in Rrp5 that frees the rRNA cleavage site and allows access for the endonuclease Rcl1 to separate the subunits (Khoshnevis et al., 2019). The DEAD-box ATPase Rok1 then pushes the assembly cascade forward by releasing Rrp5 from pre-40S to remain with the emerging pre-60S subunit (Khoshnevis et al., 2016). Thus, by coupling the progression of 40S assembly to early assembly of the 60S subunit, this checkpoint prevents the unwanted assembly of excess 40S subunits generated by premature transcription termination or rRNA degradation.

Failure to pass this checkpoint through mutations in Noc1 or Rrp5 results in a pre-18S rRNA cleavage defect in yeast and the degradation of 40S assembly intermediates (as precursor rRNA does not accumulate), leading to a severe growth defect (Khoshnevis et al., 2019). Conversely, overexpression of Rcl1 bypasses this potential QC step by permitting Rcl1 access to the rRNA cleavage site through overcrowding, even in the presence of a mutant Noc1 (Khoshnevis et al., 2019). While this bypass fully rescues the growth and rRNA cleavage defects, it remains unknown whether increased proteasomal decay of free 40S subunits obscures differences in the subunit ratio expected from QC bypass. Therefore, the Rrp5 checkpoint tests domain I assembly via a pass–fail checkpoint, but for this to be considered QC, future work is needed to verify whether skipping this step alters subunit stoichiometry or produces defective 60S subunits. Moreover, it remains unknown whether the Rok1-dependent release of Rrp5, which renders subunit separation irreversible, is dependent on correct assembly and therefore retroactively inspects assembly.

Interestingly, a nascent 60S intermediate still linked to an SSU processome-sized pre-40S intermediate has been recently recovered in yeast expressing a dominant negative mutant of the helicase Mak5 (Ismail et al., 2022). Previous work indicates a block after the separation of pre-40S and pre-60S rRNAs in this Mak5 mutant (Bernstein et al., 2006). Thus, it is possible that separation of the nascent 40S and 60S molecules requires not just the Rrp5/Rcl1-regulated cleavage step but additional 60S maturation steps. Intriguingly, additional data link early rRNA modification events in domain V to this separation step (Bhutada et al., 2022; Ismail et al., 2022; Aquino et al., 2021; Joret et al., 2018), although the details, as well as the possibility that this involves a QC checkpoint for rRNA modification or folding remain to be investigated.

#### Ribosome neck maturation (Fig. 1, step 2)

Another example of an assembly step that features aspects of QC is the exchange of Bud23 (WBSCR22 in humans) for Rio2 (RIOK2 in humans) after the ribosome neck has formed (Black and Johnson, 2022), enabling pre-40S nuclear export. Acting as a hinge during head rotation, the neck plays a crucial function for tRNA translocation during translation (Korostelev et al., 2008; Mohan et al., 2014).

Bud23 binding to Dis-C appears to precede the release of U3 by Dhr1 and is associated with the release of Utp2, Nop14, and Imp4 (Black et al., 2020), as well as Emg1, which it replaces on the subunit interface (Cheng et al., 2022). Interestingly, Bud23 also stabilizes a conformation of h18 that is both distinct to that observed in earlier pre-40S ribosomes, as well as mature 40S (Cheng et al., 2022), perhaps indicating a role in chaperoning rRNA folding. Release of U3 snoRNA and binding of Bud23 are both required for the binding of Rps2 (uS5), Rps0 (uS2), and Rps21 (eS21). Consistently, the absence of Bud23 impairs pre-40S nuclear export, leading to reduced production of mature 40S subunits (White et al., 2008) and a severe growth defect, which can be suppressed by weakly binding mutants of these AFs and overexpression of Rps2 (Black et al., 2020; Black and Johnson, 2022). Binding of Rps2, Rps0, and Rps21 in turn is required for Bud23 release (Black and Johnson, 2022) promoted by the kinase Rio2, which then takes the position of Bud23 (Black and Johnson, 2022; Linnemann et al., 2019). Surprisingly, even though Rio2, Rps2, Rps0, and Rps21 promote Bud23 release in a partially reconstituted system (Black and Johnson, 2022), Rio2 binding is independent of these RPs (Linnemann et al., 2019; Black and Johnson, 2022). Therefore, there are either two binding sites for Rio2, as recently shown for Rio1 (Ameismeier et al., 2020), or there is another unidentified step. Regardless, because Rio2 recruitment contributes to efficient nuclear export of pre-40S subunits (Zemp et al., 2009), Bud23 binding and release establish a pass–fail checkpoint for the recruitment of Rps2, Rps0, and Rps21. What remains to be seen is whether the suppressor mutants alone display incomplete assembly of Rps0, Rps2, and Rps21, or misfolded h18 as would be expected if this system is a bonafide QC mechanism.

### Cytoplasmic pre-40S assembly overview

**Figure 2.**
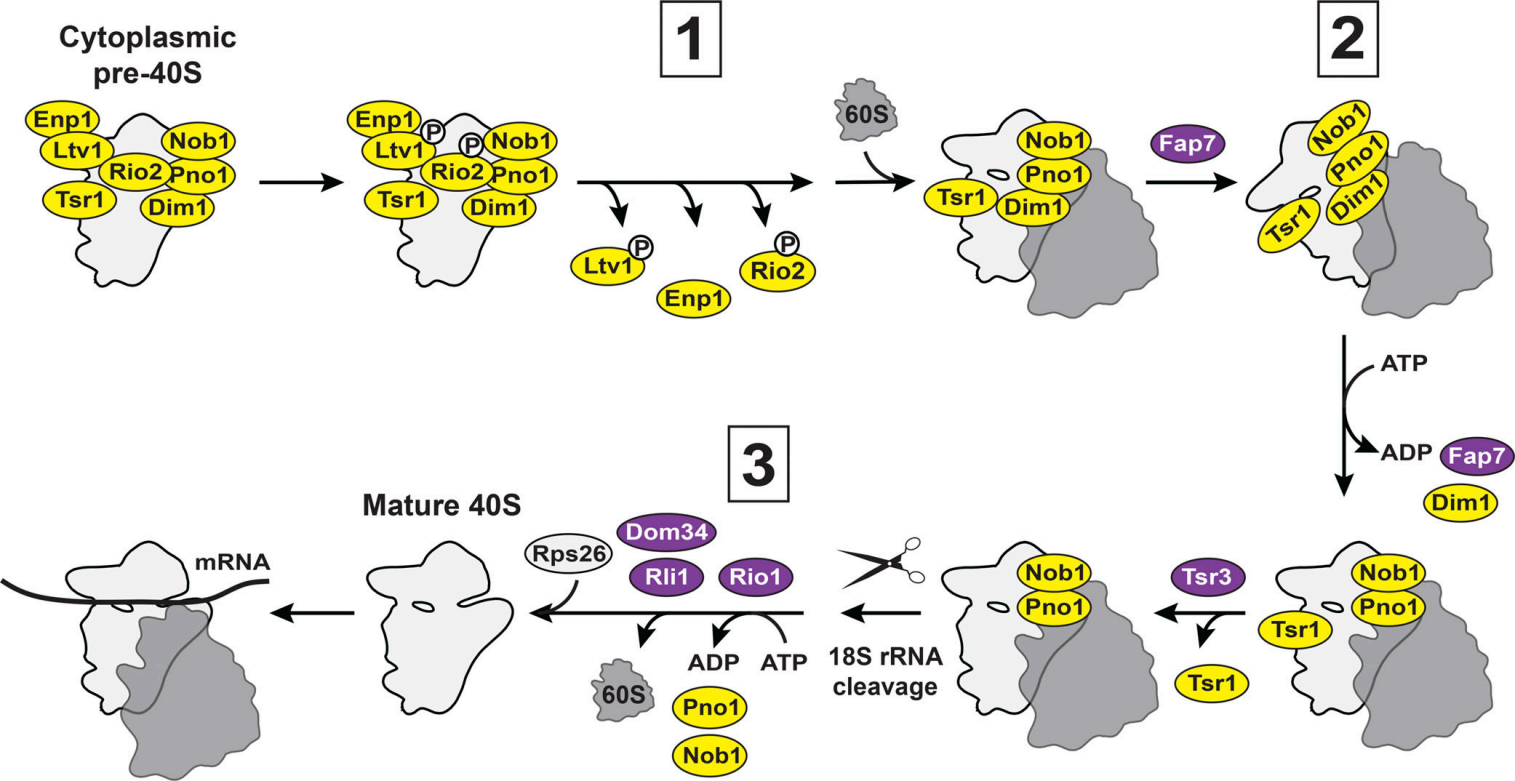
**Cartoon of cytoplasmic pre-40S assembly.** Numbers 1–3 represent three QC steps: (1) testing scanning and translation initiation, (2) evaluation of translocation, and (3) inspection of 18S rRNA cleavage. Described in more detail in the section titled “Cytoplasmic pre-40S assembly overview.”

The pre-40S ribosome that is exported to the cytoplasm contains pre-18S rRNA that requires 3′-end maturation (termed 20S or 18S-E pre-rRNA in yeast or humans, respectively), most of the RPs found in the mature 40S, and is bound to seven AFs (Ltv1, Enp1, Rio2, Dim1, Tsr1, Nob1, and Pno1) that block 60S subunit joining, the binding sites for translation initiation factors and tRNA, and obstruct the mRNA channel (Strunk et al., 2011; Heuer et al., 2017; Scaiola et al., 2018; Johnson et al., 2017; Ameismeier et al., 2018; Ameismeier et al., 2020; McCaughan et al., 2016; Fig. 2). This is the most stable assembly intermediate in yeast and is committed to the cytoplasmic assembly cascade by Hrr25(CK1δ in humans)-dependent phosphorylation of Ltv1 (Schäfer et al., 2006; Ghalei et al., 2015; Mitterer et al., 2016) to stimulate the ordered release of Ltv1, Enp1 (BYSL in humans), and Rio2 (Ghalei et al., 2015; Huang et al., 2020). This finalizes 40S head assembly via rRNA folding, re-positioning of Rps3 (uS3), and incorporation of Rps10 (eS10) and Asc1 (RACK1 in humans; Huang et al., 2020; Zemp et al., 2014; Ghalei et al., 2015; Schäfer et al., 2006; Huang and Karbstein, 2021; Mitterer et al., 2016; Mitterer et al., 2019). The translation initiation factor eIF5B then binds the pre-40S subunit to promote 60S subunit joining, forming an 80S-like ribosome that is not bound to mRNA or tRNA and will not produce protein (Strunk et al., 2012; Lebaron et al., 2012; Rai et al., 2021). Prior to or during this transition to 80S-like ribosomes, Tsr1 and Dim1 reposition, priming Dim1 for release (Rai et al., 2021). The ATPase Fap7 binds 80S-like ribosomes (Strunk et al., 2012; Ghalei et al., 2017; Granneman et al., 2005), taking advantage of the unfolded and opened platform of 80S-like ribosomes compared with mature 80S ribosomes (Rai et al., 2021) to induce a structure resembling the rotated state, an essential intermediate during translocation, resulting in Dim1 release (Ghalei et al., 2017). The release of Dim1 repositions the 3′-end of the 18S rRNA (Johnson et al., 2017; Ameismeier et al., 2018; Ameismeier et al., 2020; Rai et al., 2021) to the Nob1 active site (Lamanna and Karbstein, 2011; Lamanna and Karbstein, 2009; Pertschy et al., 2009), thereby licensing rRNA processing. Around the time of Fap7-dependent release of Dim1, Tsr3 binds 80S-like ribosomes to add an amino-carboxypropyl (acp) group to 18S:U1191 (U1248 in humans; Hector et al., 2014; Meyer et al., 2016; Huang et al., 2022). Tsr3, whose binding site overlaps those of Rio2 and Rio1 immediately before and after, respectively, is released after modifying the rRNA, thus allowing the ATPase Rio1 to bind pre-40S (Huang et al., 2022) and remove Nob1 and Pno1 following 3′-end formation of 18S rRNA by Nob1 (Parker et al., 2019; Ameismeier et al., 2020; Plassart et al., 2021; Widmann et al., 2012; Belhabich-Baumas et al., 2017; Turowski et al., 2014). 80S-like ribosomes are then separated by the translation termination factors Dom34 and Rli1 (Strunk et al., 2012). Rps26 (eS26), whose binding was blocked by Pno1, is incorporated into the now mature, translationally competent 40S ribosome (Ameismeier et al., 2020; Strunk et al., 2011; Belhabich-Baumas et al., 2017; Heuer et al., 2017).

### QC steps of the translation-like cycle during 40S assembly

Due to its similarities to translation, the late cytoplasmic assembly steps involving the formation, rotation, and disassembly of 80S-like ribosomes are collectively referred to as the “translation-like cycle” (Strunk et al., 2012). While no protein is synthesized, the translation-like cycle represents a series of QC steps to confirm that nascent 40S subunits are functional prior to their first round of translation (Strunk et al., 2012). Three known QC mechanisms test the subunit’s ability to scan along the mRNA to identify the start codon (Huang et al., 2020), to translocate (Ghalei et al., 2017), and to confirm the accuracy of the 18S rRNA 3′-end (Parker et al., 2019; Parker et al., 2022 *Preprint*).

#### Examining scanning and translation initiation (Fig. 2, step 1, and Fig. 3)

**Figure 3.**
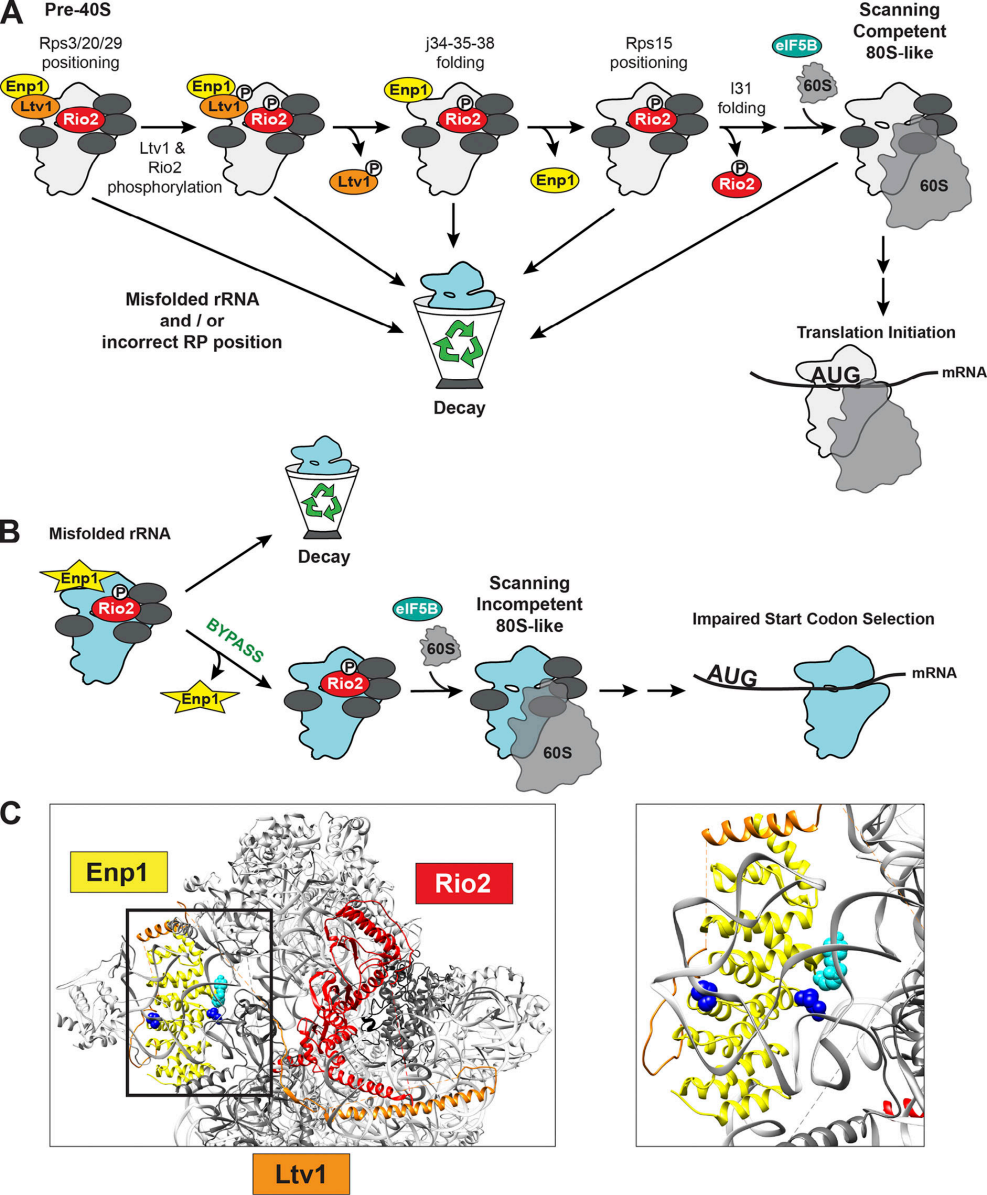
**Schematic of QC step testing scanning competence of pre-40S ribosome. (A)** QC mechanisms check for proper incorporation of RPs and folding of 18S rRNA in the ribosomal head before forming the scanning competent 80S-like ribosome. Failure to pass QC results in turnover of these pre-40S intermediates. **(B)** On the other hand, bypass of QC by using a weakly binding Enp1 mutant (yellow star) allows pre-40S with misfolded rRNA and mispositioned RPs to form 80S-like ribosomes that are eventually released into the translating pool, where they will have defects in start codon selection (Huang et al., 2020). **(C)** Structure of the human pre-40S ribosomal subunit (gray) bound by BYSL (yellow), LTV1 (orange), and RIOK2 (red; PDB accession no. 6G18; Ameismeier et al., 2018). Colored spheres indicate residues in BYSL (Enp1) that are mutated in cancer and are predicted to bypass QC (dark blue spheres indicate point mutations in residues R303 and P318, while cyan spheres mark nonsense mutations at Y265 and R267 that produce truncated Enp1 protein). Inset on the left has RPs removed for clarity (TCGA www.cancer.gov/tcga, cBioPortal www.cbioportal.org).

After nuclear export, the sequential release of the AFs Ltv1, Enp1, and Rio2 requires allosteric changes that rely on the correct positioning of Rps3 (uS3), Rps15 (uS19), Rps20 (uS10), and Rps29 (uS14) and correct folding of the three-way junction between helices h34-h35-h38 (j34-35-38; Huang et al., 2020; Huang and Karbstein, 2021; Mitterer et al., 2016; Mitterer et al., 2019; Fig. 3 A). Thus, the timed release of Ltv1, Enp1, and Rio2 requires proper head assembly. Moreover, RP misincorporation, head rRNA misfolding, or the continued binding of Rio2 prevent the formation of 80S-like ribosomes (Huang et al., 2020; Huang and Karbstein, 2021), leading to degradation of stalled intermediates. Since 80S-like ribosomes resemble the scanning complex during translation initiation (Huang et al., 2020; Rai et al., 2021), the formation of 80S-like ribosomes (which depends on release of Ltv1, Enp1, and Rio2) is a pass–fail checkpoint that not only requires the structural integrity of the ribosomal head but also tests the ability to adopt the conformation of the scanning complex (Huang et al., 2020). Bypassing this QC step (through the unregulated release of Enp1 with the Enp1_R333E mutation, deletion of an internal loop in Tsr1, Tsr1_Δloop, or a cancer-associated mutation in Rps15, Rps15_S136F) impairs start codon selection (Fig. 3 B; Huang et al., 2020), as expected from weakening the stability of the scanning complex. Therefore, this mechanism tests functionality with a pass–fail system to filter out misassembled ribosomes that persist upon QC bypass, and therefore meets all our criteria for QC. Importantly, none of these bypass mutations (Enp1_R333E, Tsr1_Δloop, or Rps15_S136F) produce growth defects or errors in ribosome assembly, thus ruling out the possibility that the observed phenotypes arise from reduced ribosome numbers (Huang et al., 2020). Moreover, two of these mutations are in AFs, which are not present in mature ribosomes, suggesting that the fidelity phenotypes shared with Rps15_S136F arise from bypassing assembly QC.

The C-terminal tail of Rps15 is mutated in 20% of all chronic lymphocytic leukemia patients (Bretones et al., 2018; Ljungström et al., 2016). In yeast, these cancer-associated mutations do not demonstrate assembly blocks or growth phenotypes, but one (Rps15_S136F, corresponding to Rps15_S139F in humans) bypasses the checkpoint for h31 folding, thereby producing ribosomes defective in start-codon selection (Huang et al., 2020). Defective start codon recognition leads to global translational reprogramming in yeast (Zhou et al., 2020). Consistently, human cells with the neighboring Rps15_P131S mutation have altered the translation of 133 mRNAs (Ntoufa et al., 2021; this mutant was not tested for its ability to bypass QC). Moreover, the yeast QC suppressor mutant Enp1_R333E (Huang et al., 2020) is found analogously in cancer patients (BYSL-R303W or R303Q, The Cancer Genome Atlas [TCGA] www.cancer.gov/tcga, cBioPortal www.cbioportal.org; Fig. 3 C), supporting the notion that QC bypass occurs within cancer cells. Nonetheless, given its location in the P-site (Simonetti et al., 2020), direct effects during translation arising from the Rps15 mutations cannot be ruled out and might additionally contribute to the cancer phenotype.

#### Cancer cells exploit leaky checkpoints

Ribosomes from yeast and human cells lacking Ltv1 have misfolded head rRNA, mispositioned Rps3 (uS3), and reduced levels of Rps10 (eS10) and Asc1, leading to growth defects, stress resistance, and impaired 18S non-functional rRNA decay (Collins et al., 2018), which otherwise targets defective mature 40S subunits for degradation (LaRiviere et al., 2006; Cole et al., 2009; Limoncelli et al., 2017; Sugiyama et al., 2019). While Ltv1 insufficiency leads to ribosomes detained by QC (due to their defect in j34-35-38 folding), this step is leaky, leading to the formation of ribosomes without Rps10 and Asc1 (likely due to mispositioned Rps3, and/or Rps20 and Rps29). Leaky QC might arise in part because checking the positioning of these proteins requires the presence of Ltv1 (Huang et al., 2020). This Achilles heel is exploited by cancer cells that downregulate Ltv1. For example, the triple-negative breast cancer cell line MDA-MB-231 has substoichiometric levels of LTV1, resulting in ribosomes with a disrupted head structure (reduced occupancy of RPS3, RPS10, and RACK1 [human Asc1]) that are defective in stop codon recognition (Collins et al., 2018). Moreover, deletion of LTV1 occurs in various cancers (TCGA www.cancer.gov/tcga, cBioPortal www.cbioportal.org), which is unusual as AFs typically increase in abundance in cancers. More generally, reduced RP stoichiometry is a common theme in cancers, especially when p53 is inactive (Ajore et al., 2017), and is associated with poor patient outcomes (Guimaraes and Zavolan, 2016; Kulkarni et al., 2017).

Similarly, Bowen-Conradi syndrome, a disorder characterized by bone marrow failure, developmental anomalies, and early infant death, is caused by a single point mutation (D86G) in the nucleolar RNA methyltransferase EMG1 (Armistead et al., 2009; Wurm et al., 2010). EMG1 is essential for the formation of the SSU processome, is part of a modification cascade producing the m^1^acp^3^Ψ modification at U1248 (Meyer et al., 2011), and is important for the proper folding of j34-35-38 (Huang and Karbstein, 2021). The disease-causing D86G mutation reduces EMG1 protein levels, rRNA methylation, and impairs 40S subunit maturation (Meyer et al., 2011; Warda et al., 2016). While the protein is essential, mutations that inactivate the protein have no effect on growth in the yeast system (Meyer et al., 2011; Leulliot et al., 2008). Thus, rather than being caused by a lack of rRNA modification, Bowen-Conradi syndrome may be caused by the absence of the EMG1 protein in the nucleolus, where it normally binds and likely stabilizes the UtpB switch (Hunziker et al., 2019). Thus, in the absence of Emg1, formation of the 90S pre-ribosome is impaired, thereby explaining the reduced ribosome numbers and cell proliferation defects underlying this ribosomopathy (Armistead and Triggs-Raine, 2014; Armistead et al., 2015; Warda et al., 2016). However, it appears that some assembly can proceed, stabilized either by redundant interactions or the absence of Nop6 (Buchhaupt et al., 2007; Schilling et al., 2012). The resulting ribosomes are misfolded in j34-35-38 (Huang and Karbstein, 2021; and perhaps elsewhere), and therefore are partially detained in the same (leaky) cytoplasmic scanning QC step that detains ribosomes from Ltv1-deficient cells (Huang et al., 2020; Huang and Karbstein, 2021). Given that this leaky checkpoint involves two proteins that are not essential in yeast, it is tempting to speculate that rendering them non-essential is a way to produce a small number of ribosomes lacking RPS10 and RACK1, or the Emg1-dependent methylation, when necessary.

Faulty ribosomes and reduced ribosome numbers may also work in tandem to cause disease. For one, reduced ribosome numbers decrease ribosome collisions (Simms et al., 2017; Zhao et al., 2021), and are therefore expected to stabilize translating dysfunctional ribosomes (Parker et al., 2022 *Preprint*). Moreover, reduced ribosome numbers can also differentially affect translation of individual mRNAs (Lodish, 1974; Mills and Green, 2017; Ferretti and Karbstein, 2019; Khajuria et al., 2018; Ivanov et al., 2022), thereby further contributing to disease.

#### Testing translocation (Fig. 2, step 2, and Fig. 4)

In addition to selecting the correct start site and amino acid, the small subunit is responsible for maintaining the reading frame during translocation, a process where the mRNA–tRNA pairs move through the ribosome to enable binding of the next tRNA. Translocation is facilitated by conformational changes in the 40S subunit, where the 40S subunit body first rotates relative to the 60S subunit followed by a pivot of the 40S head (Munro et al., 2009). Correct translocation is critical for reading frame maintenance, and even small disturbances in this process can lead to frameshifting (Caliskan et al., 2014; Chen et al., 2014), indicating the importance of releasing only translocation-competent 40S ribosomes into the translating pool.

**Figure 4.**
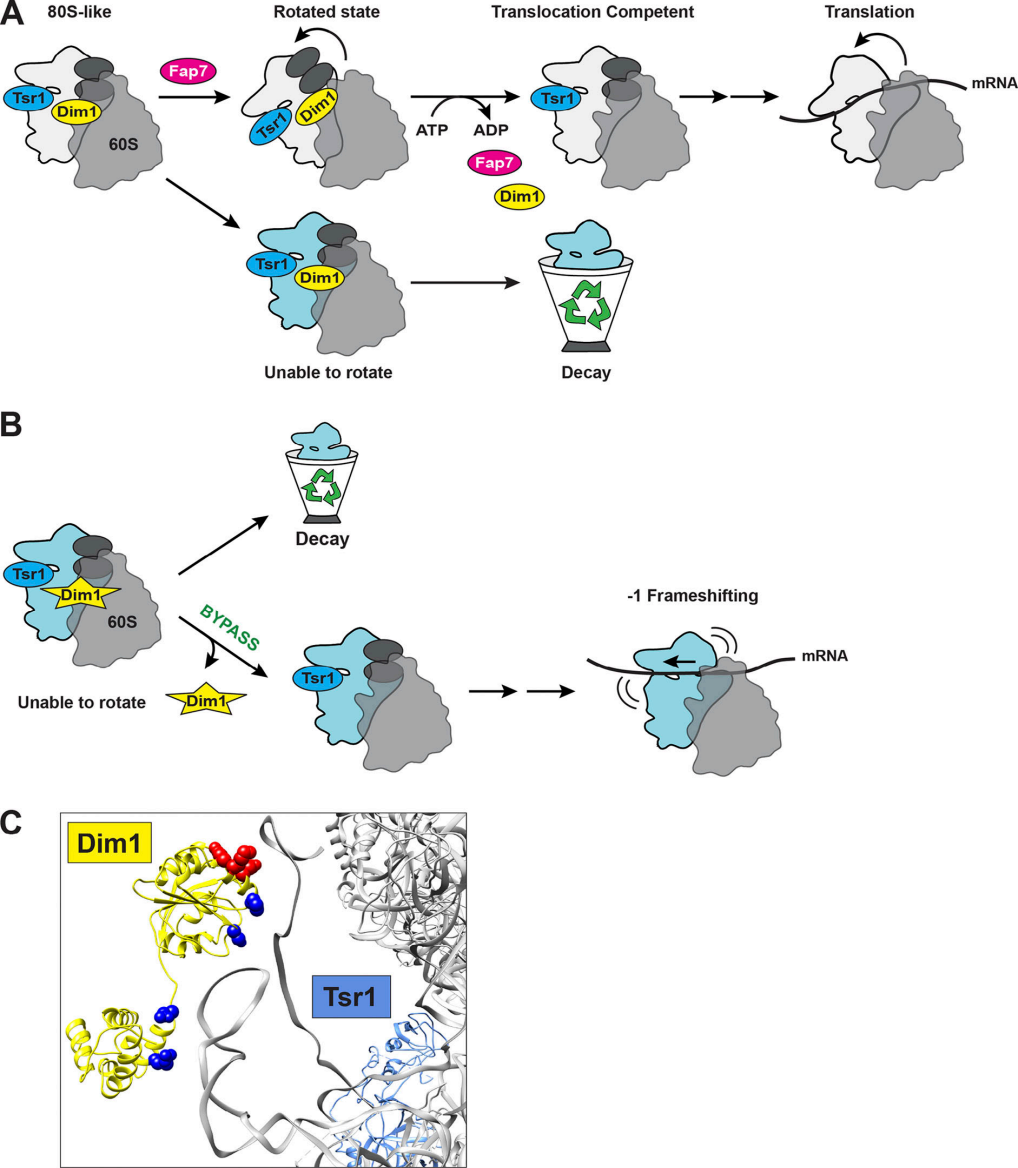
**Schematic of Fap7-mediated QC step verifying translocation capability of the pre-40S subunit. (A)** Fap7 releases Dim1 to allow pre-40S to continue their assembly only after confirming that the pre-40S can form the rotated state needed to maintain the reading frame during translation. Failure to pass this QC results in turnover of 80S-like intermediates. **(B)** Bypass of this QC using a weakly binding Dim1 mutant (yellow star) allows 40S subunits to continue maturation and causes −1 frameshifting during translation (Ghalei et al., 2017). **(C)** Structure of the 80S-like ribosome (gray, 60S is omitted for clarity) bound by Dim1 (yellow) and Tsr1 (blue; PDB accession no. 6WDR; Rai et al., 2021). Dim1_EKR (E93/K96/R97) is shown in red spheres. Dark blue spheres indicate residues in DIMT1 (P88A, P88T, D113N, N219T, and R228M) that are mutated in cancer and are predicted to bypass QC (TCGA www.cancer.gov/tcga, cBioPortal www.cbioportal.org).

Due to the importance of translocation, it is not surprising that the ability to carry out this critical step is quality controlled via the essential ATPase Fap7. Fap7 binds 80S-like ribosomes to induce a translocation-like rotated state and to release Dim1 (Ghalei et al., 2017; Fig. 4 A). Dim1 release results in an rRNA conformational change repositioning the 3′-end of 18S rRNA to the Nob1 active site to promote rRNA cleavage in 80S-like ribosomes, thereby licensing the final rRNA maturation step discussed below (Ameismeier et al., 2018; Ameismeier et al., 2020; Johnson et al., 2017; Rai et al., 2021; Lamanna and Karbstein, 2009). In the absence of active Fap7, assembly is stalled and 80S-like ribosomes accumulate (Strunk et al., 2012; Ghalei et al., 2017; Granneman et al., 2005). A mutation in the 60S protein Rpl3 that stabilizes the rotated state (Rpl3_H256A) partially rescues the absence of Fap7, while a mutation that destabilizes the rotated state (Rpl3_W255C) phenocopies the absence of Fap7. Moreover, a weak-binding Dim1 mutant, Dim1_EKR, releases from 80S-like ribosomes independent of Fap7 (Ghalei et al., 2017), thereby bypassing this subunit rotation checkpoint. While Dim1_EKR partially suppresses the growth phenotype and the accumulation of 80S-like ribosomes upon Fap7 inactivation, ribosomes from cells expressing Dim1_EKR have an increased propensity toward translation fidelity errors, with a nearly threefold increase in −1 frameshifting (Fig. 4 B; Ghalei et al., 2017). Dim1_EKR has essentially no growth phenotype and does not impair Dim1’s methylation activity (Ghalei et al., 2017), suggesting that these defects arise from the Fap7 bypass and not reduced ribosome numbers or the absence of the methylation, and strongly supporting the notion that checkpoint bypass leads to dysfunctional ribosomes.

Interestingly, a mutation in human Dim1, DIMT1_E93D, similar to Dim1_EKR in yeast (Fig. 4 C), is found in cancer patients, supporting the potential of bypassed QC in cancer cells (TCGA www.cancer.gov/tcga, cBioPortal www.cbioportal.org). Moreover, mutations in the essential internal loop of Rpl10 (uL16) are found in ∼8% of pediatric T-cell acute lymphoblastic leukemia (T-ALL) patients, with Rpl10_R98S being the most common (De Keersmaecker et al., 2013). Since Rpl10_R98S stabilizes the rotated state (Sulima et al., 2014b), akin to Rpl3_H256A, it is expected to bypass the Fap7-dependent checkpoint as we have shown for Rpl3_H256A (Ghalei et al., 2017). Consistently, Rpl10_R98S increases −1 frameshifting, the same effect observed in the Dim1_EKR mutant, and has defects in termination codon recognition (Sulima et al., 2014b). Rpl10_R98S also fails to release the AF Nmd3 in the late stages of pre-60S assembly (Patchett et al., 2017), as discussed in more depth below. Bypassing this defect with a mutation in Nmd3 that weakens its binding can rescue the growth defect but not the errors in translation, indicating that it arises from faulty ribosomes, not altered ribosome numbers (Sulima et al., 2014b). While additional experiments will be required to fully test the proposal that the Rpl10_R98S mutation affects the integrity of not only the 60S (directly) but also the 40S (indirectly) via the Fap7-mediated QC step, we note that the Johnson lab has also identified a set of additional mutations in Rpl10, termed class II mutants, that have defects in both 40S and 60S assembly, as judged by polysome profiles, and which cannot be rescued by bypass mutants of 60S defects (Bussiere et al., 2012). Importantly, this class of mutants destabilizes the rotated state (Sulima et al., 2014a) and is thereby expected to phenocopy Fap7 depletion, as we have shown for Rpl3_W255C, thus providing evidence for a role of Rpl10 in 40S maturation.

Around the time of Fap7-mediated Dim1 release, Tsr3 modifies 18S:U1191 (U1248 in humans) with the acp group to complete the m^1^acp^3^Ψ modification (Hector et al., 2014; Meyer et al., 2016; Huang et al., 2022). Interestingly, this modification, located in the P-site, is substoichiometric in 45% of human colon cancer patients, and over 22 different cancers show hypomodification at this site (Babaian et al., 2020), highlighting its importance in ribosome assembly and/or function and cellular health.

Deletion of Tsr3 allows for scrambling of the order in which the kinases Rio2 and Rio1 act, leading to the release of premature ribosomes into the translating pool (Huang et al., 2022), indicating a role for the Tsr3 in assembly. However, the deletion of Tsr3 causes no growth defect in yeast or human cells (Meyer et al., 2016; Huang et al., 2022) and quantitative proteomics demonstrate an unaltered proteome in Tsr3-deleted cells (Babaian et al., 2020). Most intriguingly, Tsr3 levels are not perturbed in cancer cells that display the acp-hypomodification at 18S:U1248 (Babaian et al., 2020). Therefore, it remains unclear what causes the hypomodification and what role, if any, it plays in cancer cells. One hypothesis is that the modification is merely a checkmark reflecting successful and correct assembly. Future experiments will be required to test this hypothesis and assess which aspect of functionality is certified with this quality seal.

#### Licensing 40S subunits to translate (Fig. 2, step 3, and Fig. 5)

**Figure 5.**
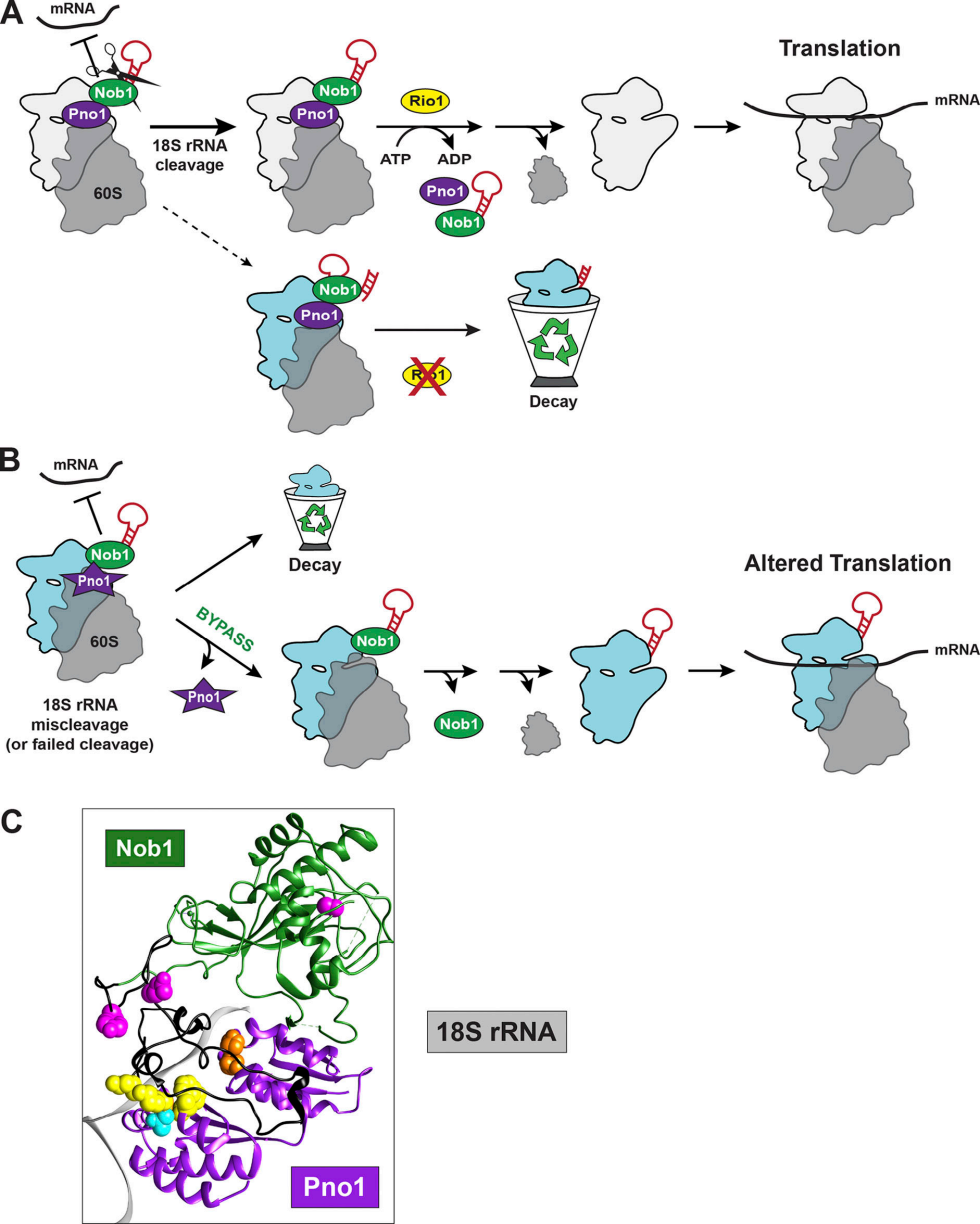
**Schematic of Rio1-mediated QC step monitoring 18S rRNA cleavage. (A)** QC mechanism regulated by Rio1 ensures only ribosomes with precise 18S rRNA cleavage are licensed to translate. The red loop on the ribosomes next to Nob1 indicates ITS1 in pre-18S rRNA. Failure to pass this QC step results in turnover of these 80S-like intermediates. **(B)** Bypassing QC via weakly binding Pno1 mutations leads to the release of ribosomes containing uncleaved 20S pre-rRNA or miscleaved 18S rRNA into the translating pool, where they cause errors in translation (20S pre-rRNA–containing ribosomes; Parker et al., 2019) or ribosome collisions (miscleaved 18S rRNA-containing ribosomes—not shown; Parker et al., 2022 *Preprint*). **(C)** Structure of the human pre-40S ribosomal subunit (3′-end of 18S rRNA is gray and RPs are removed for clarity) bound by PNO1 (purple) and NOB1 (green and black; PDB accession no. 6ZXE; Ameismeier et al., 2020). Colored spheres indicate residues in PNO1 or NOB1 that are mutated in cancer and are predicted to bypass QC. Pink spheres indicate nonsense mutations in NOB1 (C234, Y334, and Q348) and black indicates the section of NOB1 truncated after S325 (homologous to yeast Nob1_1-363; Parker et al., 2019). Mutations in PNO1 include PNO1_K186/K189/K191/F192 (homolog to yeast Pno1_KKK/F; yellow) and cancer mutations PNO1_T190N (homolog to yeast Pno1_T212N; cyan) and PNO1_R84I, P87Q, A184T (orange; TCGA www.cancer.gov/tcga, cBioPortal www.cbioportal.org).

While immature ribosomes can translate, they have fidelity defects, produce distinct stress responses, and do not support viability (Strunk et al., 2012; Soudet et al., 2010; Parker et al., 2019), indicating altered translational properties. To prevent immature ribosomes from entering the translating pool, the nuclease Nob1, which produces the mature 18S rRNA 3′-end, and its binding partner Pno1 establish a QC checkpoint regulated by the ATPase Rio1. As Nob1 blocks mRNA recruitment by pre-40S ribosomes, its release is required for the first round of translation by nascent ribosomes (Parker et al., 2019). rRNA cleavage weakens Nob1 binding, allowing for its release together with Pno1, dependent on the ATPase activity of Rio1 (Parker et al., 2019; Fig. 5 A). Rio1 also distinguishes between correctly and miscleaved rRNA, which is abundant in vitro (Pertschy et al., 2009; Lebaron et al., 2012; Lamanna and Karbstein, 2011; Veith et al., 2012) but produces only partially functional ribosomes in vivo (Parker et al., 2022 *Preprint*). Thus, this mechanism ensures that only ribosomes with correctly cleaved 18S rRNA are licensed to translate (Parker et al., 2019; Parker et al., 2022 *Preprint*). This checkpoint can be bypassed by mutations in Pno1 (Pno1_T212N [Huang et al., 2022] or Pno1_KKK/F [Parker et al., 2019]), or Nob1 (Nob1_1-363 [Parker et al., 2019]), which enable release of immature (Parker et al., 2019) or miscleaved (Parker et al., 2022 *Preprint*) ribosomes into the translating pool (Fig. 5 B), demonstrating that this is a true QC checkpoint. Importantly, neither Pno1_T212N (Huang et al., 2022) nor Nob1_1-363 (Parker et al., 2019) demonstrates growth defects, again arguing that reduced ribosome numbers are not responsible for the observed defect.

Interestingly, the homologous mutation to yeast T212N (PNO1_Y190N; Fig. 5 C) is found in cancer patient cells, akin to mutations in NOB1, PNO1, and RIOK1 (Fig. 5 C) that have the potential to disrupt the interactions between these proteins or with rRNA (TCGA www.cancer.gov/tcga, cBioPortal www.cbioportal.org), and are expected to act akin to Pno1_T121N or Pno1_KKKF. This shows that QC bypass occurs within cancer cells. Whether and how much this contributes to cancer development or progression remains to be seen.

Beyond mutations, the concentration of Rio1 also affects the stringency of this QC mechanism as correct processing affects Rio1 binding affinity, such that miscleaved (or uncleaved) RNAs are bound more weakly (Parker et al., 2022 *Preprint*). Increased Rio1 concentrations allow Rio1 to bind and act on ribosomes with unprocessed or misprocessed 18S rRNA, mistakenly releasing Nob1 and Pno1 from these ribosomes (Parker et al., 2019; Parker et al., 2022 *Preprint*). Whole-genome sequencing of cancer cells revealed that RIOK1 is frequently amplified in cancer (TCGA www.cancer.gov/tcga, cBioPortal www.cbioportal.org). Moreover, relative to mRNAs encoding RPs (to normalize for increased ribosome assembly in cancer), RIOK1 is upregulated in 29% of all cancers including ovarian cancer, melanomas, and thymic carcinomas (Parker et al., 2022 *Preprint*). While it remains unknown whether these changes in RIOK1 abundance affect cancer progression by allowing miscleaved or immature ribosomes to participate in translation, the data in yeast demonstrate that Rio1 concentration must be exquisitely controlled to render this QC step both functional and efficient and that cancer cells have disrupted this delicate control.

### Overview of pre-60S assembly

**Figure 6.**
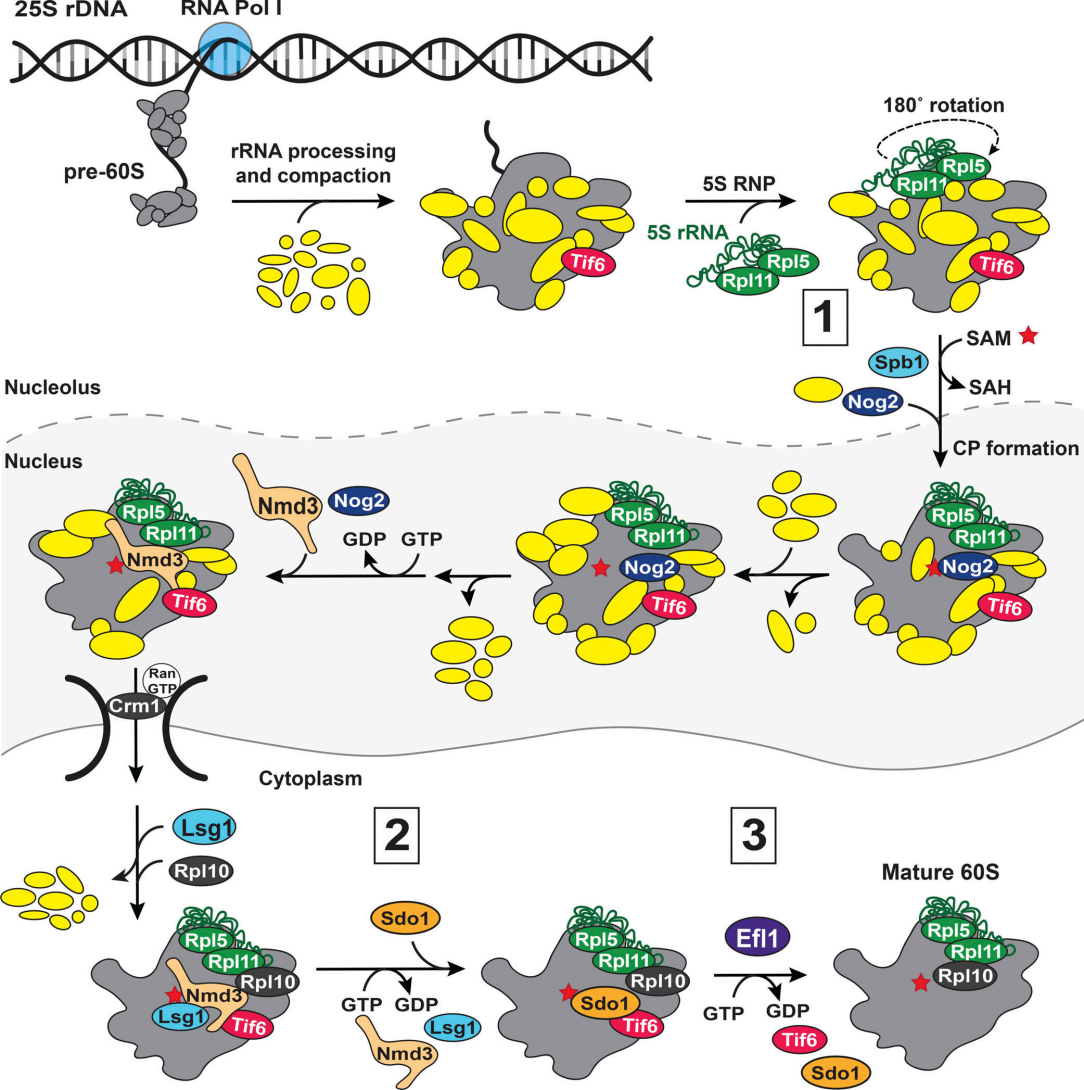
**Cartoon of pre-60S assembly.** Numbers 1–3 represent three potential QC steps: (1) inspecting A-site methylation, (2) testing PTC assembly, and (3) testing translational GTPase activation. Described in more detail in the section titled “Overview of pre-60S assembly.” The 5S RNP (consisting of the 5S rRNA, Rpl5, and Rpl11) is green and 25S:G_m_2922 methylation is represented by a red star. SAM: S-adenosyl-methionine; SAH: S-adenosyl-homocysteine.

Pre-60S biogenesis also begins co-transcriptionally with the recruitment of early AFs, which, as discussed above, orchestrate the separation of pre-40S and pre-60S subunits by cleaving between 18S and 5.8S rRNA after domain I of 25S rRNA has been transcribed (Osheim et al., 2004; Kos and Tollervey, 2010). Once transcription of the pre-rRNA is complete, the 3′-end of 25S rRNA is formed, followed by ITS1 removal to produce the 5′-end of 5.8S rRNA, and initial rRNA compaction (Sloan et al., 2013; Kempers-Veenstra et al., 1986; Henry et al., 1994; Kufel et al., 1999; Lygerou et al., 1996; Oeffinger et al., 2009; Goldfarb and Cech, 2017; Kater et al., 2017; Sanghai et al., 2018). During early nucleolar assembly, the 5S RNP, composed of the mature 5S rRNA bound to RPs Rpl5 (uL18) and Rpl11 (uL5), binds to the pre-60S (Zhang et al., 2007; Asano et al., 2015; Kharde et al., 2015; Madru et al., 2015) in an immature conformation (Leidig et al., 2014; Fig. 6). Functional centers begin to emerge in pre-60S as early acting AFs are released, coupled to rRNA structural rearrangements and the incorporation of RPs and additional AFs (Wu et al., 2016; Wilson et al., 2020). First, the polypeptide exit tunnel (PET, the channel through which the nascent polypeptide chain exits the 60S subunit and promotes initial protein folding [Wilson and Beckmann, 2011]) is formed (Bassler et al., 2010; Kater et al., 2020), followed by the removal of most of ITS2 at the 3′-end of 5.8S rRNA in a series of processing steps. The mature 3′-end of the 5.8S rRNA will be formed in the cytoplasm (Thomson and Tollervey, 2010).

Late in nuclear pre-60S assembly, the 5S RNP is fully incorporated by rotating 180° to form the central protuberance (Leidig et al., 2014). The resulting structural rearrangements of rRNA helices around the peptidyl-transferase center (PTC) stimulate the GTPase activity of Nog2, leading to the release of Nog2 and binding of the essential nuclear export factor Nmd3 (Barrio-Garcia et al., 2016; Matsuo et al., 2014).

In the cytoplasm, the export factors and remaining AFs are removed and the final RPs are incorporated into the 60S subunit in a stepwise manner (Lo et al., 2010). Toward the end of this cascade, the GTPase Lsg1 releases Nmd3 allowing Sdo1 (SBDS in humans) to bind (Hedges et al., 2005). The GTPase Efl1 is then recruited and uses its GTPase activity, stimulated by Sdo1, to release Tif6 (eIF6 in humans), Efl1, and Sdo1 (Bécam et al., 2001; Senger et al., 2001; Menne et al., 2007; Finch et al., 2011; Weis et al., 2015; Kargas et al., 2019). The 60S subunit is now mature and free to bind 40S subunits to engage in translation (Russell and Spremulli, 1979; Gartmann et al., 2010; Klinge et al., 2011). While the pre-60S assembly pathway is outlined in Fig. 6, a comprehensive review can be found here (Greber, 2016; Konikkat and Woolford, 2017; Klinge and Woolford, 2019; Bassle and Hurt, 2019).

### Quality control during pre-60S assembly

#### Inspecting A-site methylation (Fig. 6, step 1)

During nuclear pre-60S assembly in yeast, the methyltransferase Spb1 methylates G2922 of 25S rRNA at its 2′-OH (Lapeyre and Purushothaman, 2004; Hansen et al., 2002). The successful installation of this modification in the A-site is then reinspected in a subsequent step, the binding and activation of the GTPase Nog2 (Cruz et al., 2022; Yelland et al., 2023). While modified G_m_2922 engages the Nog2 active site stably (Cruz et al., 2022; Yelland et al., 2023), it does not allow for GTPase activation as the methylation blocks an essential hydrogen bonding network to the γ-phosphate, present with unmethylated G2922 (Cruz et al., 2022). Thus, G2922 leads to rapid GTP hydrolysis and thus inactivation of Nog2, which appears to mostly dissociate. In contrast, G_m_2922 allows for stable binding of Nog2. Thus, the Nog2 GTPase activity is used as a timer, which retroactively inspects G2922 methylation in a manner that allows the mistake to be fixed, as Nog2 dissociation also allows Spb1 the chance to rebind and methylate the RNA. While bypass mutations for this proofreading checkpoint have been identified (Cruz et al., 2022; Yelland et al., 2023), it remains unknown whether these produce misassembled and dysfunctional ribosomes, thus precluding us from unambiguously labeling this a QC step.

#### Testing PTC assembly (Fig. 6, step 2)

The completion of the PTC is initiated by the binding of Rpl40 (eL40), which stabilizes H89 in its mature conformation. As H89 forms one face of the Rpl10 (uL16) binding site, this allows for the recruitment of Rpl10. This in turn repositions H38, which forms the second face of the Rpl10 binding site, away from Nmd3, thereby weakening its binding and allowing the L1 stalk to be partially retracted. The change in the L1 stalk position then leads to a conformational change within Nmd3, which liberates it from the P- and E-sites and allows the P-site loop of Rpl10 to enter the P-site, where it blocks rebinding of Nmd3. Thus, the binding and accommodation of Rpl10 in the P-site promote conformational changes within Nmd3 and the nascent subunit that weaken Nmd3 binding. Nonetheless, previous genetic data have demonstrated that Nmd3 release requires the GTPase activity of Lsg1 (Hedges et al., 2005; West et al., 2005). Lsg1 and Nmd3 bind directly to each other, and Lsg1 is placed on top of Nmd3 (Weis et al., 2015; Kargas et al., 2019; Malyutin et al., 2017; Ma et al., 2017; Zhou et al., 2019), strongly suggesting that Lsg1 is released after GTP hydrolysis, which must occur before Nmd3 can dissociate. Based on the observation of a crosslink between the Rpl10 P-site loop and Lsg1, Warren and colleagues suggest that accommodation of the Rpl10 P-loop might be directly sensed by Lsg1, leading to the activation of its GTPase activity (Kargas et al., 2019). Consistently, mutations in Rpl10 affect this step (Bussiere et al., 2012; Patchett et al., 2017; Hedges et al., 2005). These mutations can be bypassed by mutations in Nmd3, which weaken Nmd3 binding (Bussiere et al., 2012; Hedges et al., 2005; Sulima et al., 2014b). Unfortunately, the effect on translation from these bypass mutants alone has not been tested, again preventing us from concluding (yet) that this is a QC step.

#### Testing translocational GTPase activation (Fig. 6, step 3)

Above, we have described how the ability to carry out structural changes that are important for translocation and reading frame maintenance is quality tested during pre-40S maturation. Here, we describe that the ability of the large subunit to carry out these transitions is also assessed during pre-60S assembly: Efl1 is homologous to the translation elongation factor eEF2, and its binding partner Sdo1 is structurally related to tRNA (Senger et al., 2001; Ng et al., 2009). Together, Efl1 and Sdo1 interact with the P-site, the PTC, the entrance to the PET, the P-stalk, and the sarcin–ricin loop (Weis et al., 2015), which houses the GTPase center, a position that could enable it to ensure the correct assembly of these critical locations and their position relative to each other. Efl1 then undergoes a Sdo1-stimulated GTPase-dependent conformational change to release both Sdo1 and the only other remaining AF, Tif6 (eIF6 in humans). Thus, cells lacking the non-essential Sdo1 or with defective Efl1 accumulate pre-60S subunits bound to Tif6, which causes a severe slow-growth phenotype (Senger et al., 2001; Menne et al., 2007; Lo et al., 2010; Finch et al., 2011). Weakly binding Tif6/eIF6 mutants or mutations in Efl1 mimicking its repositioned state can bypass the Efl1-GTPase requirement for Tif6 release, thus partially rescuing the growth defects, 60S subunit deficiency, and translational capacity in cells lacking functional Sdo1 (Senger et al., 2001; Menne et al., 2007; Finch et al., 2011; Weis et al., 2015). If or how these suppressor mutations affect 60S subunit function during translation is not known. Thus, the existing data demonstrate that Efl1 and Sdo1 couple the inspection of the structure and function of nascent subunit functional centers to license 60S subunits for translation but lack definitive data on QC. However, we note that as described above, mutations in Rpl10, including the T-ALL–associated Rpl10_R98S, impair the Lsg1-mediated release of Nmd3 (Sulima et al., 2014b; Patchett et al., 2017), which can be bypassed by additional mutations in Nmd3, which weaken its binding (Sulima et al., 2014b). These Nmd3 suppressor mutations almost entirely rescue the growth and assembly defects of these Rpl10 mutants (Sulima et al., 2014b; Hedges et al., 2005; Bussiere et al., 2012), indicating that intermediates in this strain are not appreciably detained in the subsequent Sdo1/Efl1-dependent step. This observation suggests either that this Sdo1/Efl1-mediated step is leaky (as observed for the non-essential Ltv1 and Emg1) or that it does not test the functionality of the P-site, despite its proximity. It might still test the functionality of the other critical sites in the 60S.

Failure to release Tif6 leads to Shwachman-Bodian-Diamond syndrome (SDS), a disease characterized by bone marrow failure, skeletal abnormalities, exocrine pancreatic insufficiency, and an increased risk of developing myelodysplastic syndrome (MDS) and acute myeloid leukemia (AML; Donadieu et al., 2005; Dror, 2005; Warren, 2018). SDS is caused by mutations that perturb SBDS protein (human Sdo1) binding or its conformational dynamics (Finch et al., 2011; Weis et al., 2015). Interestingly, ∼65% of patients with SDS acquire mutations in eIF6 in their blood and bone marrow cells that either destabilize eIF6 or weaken its binding to 60S subunits (Tan et al., 2021; Kennedy et al., 2021), while other patients acquire genomic deletions of regions in chromosome 20 that encode eIF6 (Valli et al., 2013; Khan et al., 2021; Tan et al., 2021; Pressato et al., 2012; Valli et al., 2019; Kennedy et al., 2021). These alterations are associated with milder hematological phenotypes and a lower risk of developing MDS and AML (Pressato et al., 2012; Valli et al., 2019; Valli et al., 2013), suggesting that hematological phenotypes are primarily caused by the continued presence of Tif6 on ribosomes, which blocks subunit joining. In addition, there is an SDS-like syndrome caused by mutations in EFL1 (Stepensky et al., 2017; Tan et al., 2018; Lee et al., 2021; Tan et al., 2019), which may similarly prevent the timely release of eIF6 from the nascent ribosome.

## Concluding remarks and future outlook

These examples highlight the importance of QC, as well as the work left to further describe QC mechanisms during ribosome assembly. In particular, the demonstration that QC bypass leads to malfunctioning or misassembled ribosomes is often missing, no doubt, because it can be difficult to address experimentally. For one, given the many overlapping QC pathways, bypass of one will only produce small populations of misassembled ribosomes, which can be difficult to detect in the current reporter assays for translational fidelity and maybe even harder to detect using structural probes that inspect RNA folding or RP composition. Moreover, the redundancy of QC pathways may also hide defects arising from bypass. For example, as described above, cells compensate to maintain the proper ratio of 40S/60S subunits (Gregory et al., 2019; Cheng et al., 2019), which could mask defects from disruption of mechanisms maintaining this ratio. In addition, we have recently provided evidence that functionally compromised but actively translating 40S ribosomes are cleared after being released into the translating pool via a collision-dependent pathway (Parker et al., 2022 *Preprint*). This is perhaps not surprising as non-functional ribosomes are already known to be rapidly degraded, although apparently during initiation (LaRiviere et al., 2006; Cole et al., 2009; Limoncelli et al., 2017; Sugiyama et al., 2019). While this pathway may be a major contributor to ensuring the integrity of the ribosome pool, as it is set up to detect and clear small amounts of dysfunctional 40S ribosomes, it has important implications for our ability (and our limitations) to detect misassembled 40S ribosomes. Moreover, the observation that ribosomes with different abilities to elongate could be prone to collisions, as well as the effort cells spend on QC to ensure ribosome homogeneity, as outlined herein, also suggests that functional ribosome heterogeneity may be limited to specialized cases.

As described herein, work over the last decade has provided exciting insights into QC during and even after ribosome assembly. What remains unknown is how ribosome assembly intermediates, once identified as misassembled, are degraded. Future work will hopefully delineate roles for RNA and protein degradation machineries in this process.

Finally, considering that the construction of ribosomes consumes many cellular resources (Warner, 1999), it seems wasteful for cells to degrade all misassembled ribosomes, especially late in the process. Therefore, it is tempting to contemplate whether QC involves a proofreading ability to repair previous assembly mistakes, as suggested here for the Nog2-dependent proofreading of the Spb1-dependent methylation of G2922. Indeed, our data on Rio1-dependent QC of the accuracy of 18S rRNA cleavage also indicate that Nob1 and Pno1 may be retained on miscleaved, elongated 18S rRNAs, thus allowing Nob1 a second chance to correctly cleave the rRNA (Parker et al., 2022 *Preprint*). We have also previously shown that correct folding of j31-34-35 is given a second chance (Huang and Karbstein, 2021). Similarly, missing late-binding RPs could be incorporated in a second attempt if the rejected assembly intermediates are reshuttled into the assembly cascade.

## Footnotes

1 Both our lab and the Schneider lab have demonstrated the ability of recombinant Rcl1 to separate 40S and 60S rRNAs at the correct site in vitro (Wells et al., 2016; Khoshnevis et al., 2019; Horn et al., 2011). In addition, our recombinant Utp24 is able to cleave adjacent to the 5′-end of 18S rRNA (Wells et al., 2016; Khoshnevis et al., 2019), consistent with genetic data (Bleichert et al., 2006). In contrast, the same Utp24 preparation, which is purified over three columns, does not separate 18S from 25S rRNAs, while the His-tag purified Utp24 from the Schneider lab does. Notably, this preparation does not have the ability to cleave near the 5′-end of 18S rRNA. These differences have led to the suggestion that maybe Utp24 could be an additional or an alternative nuclease to separate pre-40S and pre-60S rRNAs. While the structures do not help in resolving this issue, as the two proteins seem to dissociate simultaneously (Cheng et al., 2022), the identity of the nuclease has no implication for the discussion here, as Rcl1 might affect this step either directly (as we suggest) or indirectly, but in either case is regulated by Rrp5/Noc1/Noc2.
